# Inter-nucleosomal potentials from nucleosomal positioning data

**DOI:** 10.1140/epje/s10189-022-00185-3

**Published:** 2022-04-11

**Authors:** Kunhe Li, Nestor Norio Oiwa, Sujeet Kumar Mishra, Dieter W. Heermann

**Affiliations:** 1 Institute for Theoretical Physics, Heidelberg University, Philosophenweg 19, D-69120 Heidelberg, Germany; 2 Department of Basic Science, Universidade Federal Fluminense, Rua Doutor Sílvio Henrique Braune 22, Centro, Nova Friburgo, 28625-650 Brazil; 3 Center for Computational Biology and Bioinformatics, School of Computational and Integrative Sciences (SCIS) Jawaharlal Nehru University, New Delhi, India

## Abstract

No systematic method exists to derive inter-nucleosomal potentials between nucleosomes along a chromosome consistently across a given genome. Such potentials can yield information on nucleosomal ordering, thermal as well as mechanical properties of chromosomes. Thus, indirectly, they shed light on a possible mechanical genomic code along a chromosome. To develop a method yielding effective inter-nucleosomal potentials between nucleosomes, a generalized Lennard-Jones potential for the parameterization is developed based on nucleosomal positioning data. This approach eliminates some of the problems that the underlying nucleosomal positioning data have, rendering the extraction difficult on the individual nucleosomal level. Furthermore, patterns on which to base a classification along a chromosome appear on larger domains, such as hetero- and euchromatin. An intuitive selection strategy for the noisy optimization problem is employed to derive effective exponents for the generalized potential. The method is tested on the *Candida albicans* genome. Applying *k*-means clustering based on potential parameters and thermodynamic compressibilities, a genome-wide clustering of nucleosome sequences is obtained for *C. albicans*. This clustering shows that a chromosome beyond the classical dichotomic categories of hetero- and euchromatin is more feature-rich.

**Supplementary Information:**

The online version contains supplementary material available at 10.1140/epje/s10189-022-00185-3.

## Introduction

The organization of a complex system such as the nucleosome organization and with it the three-dimensional organization of a chromosome is influenced by hundreds of factors from DNA sequence, nucleosome remodelers to transcription factors [1]. Each of these factors influences not only the chemical environment but also the mechanical properties of the chromatin fiber such as the bending rigidity. Since the chromatin fiber is a heteropolymer, the bending rigidity is not a constant along the backbone [2]. Changing the bending rigidity by a more compact packing of the nucleosomes, for example, by a microphase separation [3, 4] changing the order parameter and packing, has an influence on the loop structure of a chromosome and hence on regulation [5].

It has long been speculated that there must be something like a mechanical code (a comprehensive map determining shapes of DNA and mechanical properties) on top of the genetic code [6, 7]. This mechanical code stems from the organizational structure of the nucleosomes since elasticity is a direct result of interatomic interaction. A tighter packing gives rise to more steric repulsion and hence higher bending rigidity. This in turn leads a reduced possibility for distal interactions, i.e., looping, hence controlling the three-dimensional organizational structure. And, there is more and more evidence surfacing that there is a richer variety of compactification of nucleosomes beyond the hetero- and euchromatin picture [8–10]. Experimental as well as theoretical work has indicated that indeed there is more than just two [11, 12].

In this work, we take the point of view that we can extract larger nucleosomal structure from nucleosomal positioning data by coarse graining.

To reveal the thermodynamic properties and hence give indication on the mechanical code, we move to a larger global scale and ask for nucleosomal distribution patterns along a single chromosome as well as universal pattern between all chromosomes of a given genome. For this, we need to eliminate some of the smaller structures to reveal structure on a coarser level which is also more in line with the local phase separation picture [13].

There are at least two main directions that can be chosen. Physically, it is possible to start with geometric properties, e.g., the bending rigidity or stiffness, which is already verified to have a significant correlation with the compaction [14, 15]. Chemically, it is desirable to extract the effective pair-wise potential between single nucleosomes, and essential properties can be calculated subsequently. This allows to compute thermodynamic properties such as the compressibility for all of stretches showing a particular pattern of nucleosome distribution. Eventually, this leads to information on the mechanical properties since it allows to bring in line information on varying compressibilities and along the chromosomes with effective potentials. Furthermore, it also allows to extract the $\chi$-parameter for the Flory–Huggins theory and shed light on the possible thermodynamic state, in particular the microphase separation [16].

## Methods

### Computational methods

One of the basic techniques to measure the nucleosome activity is the micrococcal nuclease digestion with deep sequencing (MNase-seq) [17]. The method measures the nucleosome occupancy by measuring the frequency of nucleosome-bounded DNA fragments. However, it does not directly identify the nucleosome position, the probabilistic genomic position where each nucleosome is located. In order to map the MNase-seq data to nucleosome positioning data, several programs were developed, such as NPS [18], nucleR [19], DANPOS [20], and iNPS [21] (improved nucleosome positioning from sequencing).

Our starting point is iNPS data for *Candida albicans*. The raw data (MNase-seq) are available from the Gene Expression Omnibus (GSM1542419) [22] and were measured by Puri et al. [23]. We also accessed the processed iNPS data in the NucMap database by Zhao et al. [24].

**Fig. 1:**
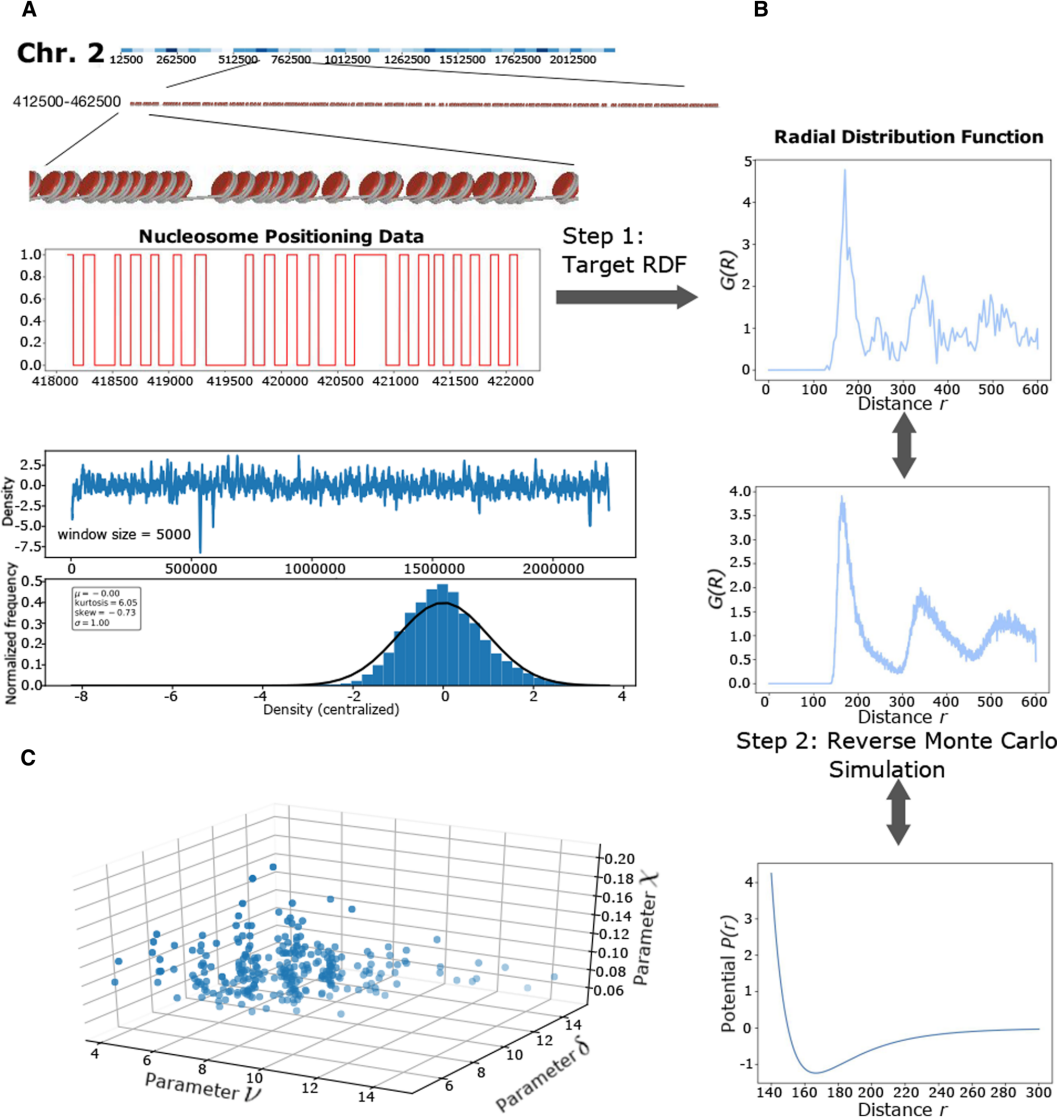
*Steps to derive inter-nucleosomal potentials from nucleosomal positioning data.* Panel **A** shows schematically the distribution of nucleosomes in a section of chromosome 2 of *C. albicans*. We split the chromosome into sections, typically of size 50,000 bp. The lower part of Panel **A** shows the density after applying a rolling mean averaging with window size 5000 bp, and the typical section size is chosen to be 10 times of this scale. Step 1 takes the red binary data. Based on this data, the radial distribution function (RDF) is computed. This step enables us to obtain a coarse-grained representation of the chromosome that allows for an effective and efficient simulation of a chromosome. There is also a 12,500 bp extra intersection at each end with its neighbor. This resolves the boundaries between the sections. Once the radial distribution is computed, we apply a cut-off to the potential. Using a reverse Monte Carlo simulation, we estimate a potential from the RDF. We employ an intuitive selection strategy, i.e., a noisy optimization technique to find the best fit for the generalized Lennard-Jones exponents (see Panel **C**)

A section of the raw data is shown in Fig. 1 in panel A indicated by the red line. The areas with value 1 are the nucleosome positions, and the areas with value 0 are voids. This data are noisy due to missing data. Furthermore, on this small scale it is difficult to discern structure.

The goal is to extract potentials from the nucleosomal positioning data. One approach to obtain those is to compute the radial distribution function (RDF) *G*(*r*) with respect to the distance *r* (measured in base pairs)

1 $$\begin{aligned} G(r) = \frac{1}{\rho N S_d}\sum _{i=1}^{N}\sum _{j=1,j\ne i}^{N}\delta (r-r_{ij}) \end{aligned}$$

where $\rho$ is the density, *N* is the number of nucleosomes, $S_d$ is a dimensional related term, $r_{ij}$ is the distance between two nucleosomes *i* and *j*, and $\delta (r-r_{ij})$ is equal to 1 if $r=r_{ij}$ and 0 otherwise.

A chromosome is split into sections of 50,000 bp with 12,500 bp extra intersection at each end with its neighbor. For each section, we calculate the corresponding RDF. The sectioning of the chromosome is such that a substantial overlap between neighboring sections is guaranteed. Thus, the actual boundary position is somewhat fuzzy so that the actual starting position becomes less relevant.

To derive pair potentials from the nucleosomal distribution patterns [25], there are several paths such as the Berg–Harris method [26], Yvon–Born–Green equation [27], and reverse Monte Carlo [28]. We employ an reverse process on the nucleosomal radial distribution function. Its solution is guaranteed to converge by combining the noisy optimization [29, 30] with the coarse-graining technique of molecular models, i.e., the reverse Monte Carlo [31, 32], and, for example, implemented for the aqueous NaCl solution [28]. We implemented the basic idea with several improvements: most importantly, a generalized Lennard-Jones model for the potential and an intuitive selection strategy (ISS) for the noisy optimization problem are used.

The reverse Monte Carlo (RMC) method is a double loop nested Monte Carlo (MC) simulation. In the inner loop, a standard molecular Monte Carlo simulation is implemented to obtain the desired parameter for a given potential, while for the outer loop a Monte Carlo Markov Chain (MCMC) [33] is employed. A MCMC step proposes a new potential, runs the inner step, compares the computed parameter with the target result, and updates the potential until the tolerance level is reached. The RMC method succeeded in many cases, for example, in NaCl solutions [28]. However, it has the flaw that it has no guarantee to convergence, especially for a complex system. This issue also emerged applying RMC for the nucleosome system. In this circumstance, we have developed two improvements.

The original RMC uses a general potential. This, however, leads to convergence problems. From the computed radial distribution function *G*(*r*) (Figure S2) and the related mean-field potential

2 $$\begin{aligned} P_{\text {MF}}(r)\propto -\log (G(r)) \; , \end{aligned}$$

we can actually observe that the target potential has a type similar to a Lennard Jones potential. Hence, without losing most of the generality, our ansatz is a generalized Lennard-Jones potential

3 $$\begin{aligned} V(r) = 4\epsilon \left[ \left( \frac{\sigma }{r}\right) ^\delta - \left( \frac{\sigma }{r}\right) ^\nu \right] \; . \end{aligned}$$

Consistent with the Lennard Jones potential, $\epsilon$ determines the amplitude, and $\sigma$ determines the length scale. The parameters $\delta$ and $\nu$ are the exponents that determine the shape and allow it to preserve most of the generality.

Another modification is substituting the MCMC step in RMC. The MCMC step is intended to solve the optimization problem, i.e., finding the RDF minimizing the differences. However, calculating an RDF from a potential via simulation produces non-negligible noise, especially for a more complex system. Therefore, the MCMC or other methods, e.g., Hill Climbing, Gradient Descent, and Simulated Annealing, have low efficiency or are not converging. Consequently, we use for this non-trivial step a noisy optimization technique (dynamic optimization [30], or optimization with erroneous oracles [34]). The straightforward application is via an evolution strategy [29]. We have modified this to an intuitive selection strategy (ISS). This approach is more stable and well suited for parallel computing. Due to this parallelization, the computational cost is strongly reduced.

The ISS is very straightforward: 1. Execute the MC simulation for each possible potential in low precision, i.e., smaller number of MC steps. 2. Choose the best *N* candidates according to a selection ratio $\theta$. 3. Increase the number of MC steps to a larger value and repeat the process. Repeating this many times, finally, there will be only one candidate, which is the result.

Note that our model is continuous along the section axis. Hence, basepair preferences of the nucleosomes are not taken into account. To include this, a modified continuous model with preferred attraction sites would be needed or a discrete model on the level of basepairs, since nucleosomes can slide as well as the uncertainty of the data has guided us in our model choice.

### Compressibility

We compute the reduced isothermal compressibilities $\chi _T^\infty$ by the block density distribution method [35, 36]. In this method, the whole section with size $L_0$ is separated into $M_b$ blocks. The size of each block is $L=L_0/M_b$. Let *N* be the number of the nucleosomes in a block. If the distribution of *N* is $P_{L,L_0}(N)$, its *k*th moments $\langle N^k\rangle _{L,L_0}$ is given by

4 $$\begin{aligned} \langle N^k\rangle _{L,L_0}=\sum _N N^k P_{L,L_0}(N) \; . \end{aligned}$$

The summation is over all possible value of *N*. Then, the reduced isothermal compressibility of a block is

5 $$\begin{aligned} \chi _T(L,L_0)=\frac{\langle N^2\rangle _{L,L_0}-\langle N\rangle _{L,L_0}^2}{\langle N\rangle _{L,L_0}} \; . \end{aligned}$$

The difference between the finite size $\chi _T(L,L_0)$ and the thermodynamic limit $\chi _T^\infty$ is related to boundary effects associated with the finite-size of the subdomains. It takes the form:

6 $$\begin{aligned} \chi _T(L,L_0\rightarrow \infty )=\chi _T^\infty +\frac{c}{L}+O\left( \frac{1}{L^2}\right) \; . \end{aligned}$$

Here *c* is a constant. Under this circumstance, the reduced isothermal compressibility of block $\chi _T(L, L_0)$ can be extrapolated to compute the reduced isothermal compressibility $\chi _T^\infty$ by just taking the limits $L, L_0\rightarrow \infty$. Hence, in the $\chi _T(L,L_0)$ vs. $M_b$ plot, the value at $M_b=0$ is the result $\chi _T^\infty$.

The block density distribution method can compute the compressibility efficiently, but the calculation needs a large amount of conformations. In this paper, after the effective potential is obtained, we generate conformations through a MC simulation of 1,000,000 MCSs for each section.

### Parameters

For the each of the eight chromosomes of the genome, we partitioned the chromosome in sections of 50,000 bp length each. There is a 12,500 bp extra intersection at each end with its neighbor to reduce the boundary effect. Thus, the total length of each section is 75,000 bp including the overlap. For the particle-based Monte Carlo simulation, section *i* starts from $12,500+50,000\cdot i$ bp to $12,500+50,000(i+1)$ bp, while actually the data are taken from $50,000\cdot i$ bp to $50,000\cdot i+75,000$ bp. This binning is applied to the whole genome. For example, the length of chr. 2 is 2.231.883 bp [37], and it is separated into 44 sections.

In the one-dimensional Monte Carlo simulation, each monomer represents a nucleosome and occupies a volume equal to the averaged nucleosome length for that section. For every MC step, a random move for each monomer is proposed. It ranges from 0 to $\lambda$. The move is rejected or accepted according to the energy difference multiplied by the Boltzmann factor $k_B T$. In our simulation, $k_B T$ is set to be 1.

The value of $\lambda$ is chosen to be the smallest value that allows the acceptance rate to be equal to or smaller than 50% on average.

**Fig. 2:**
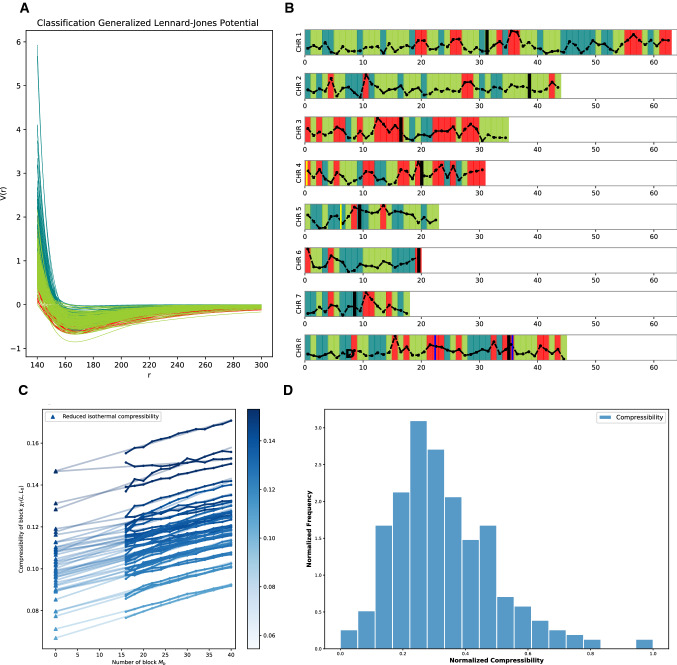
*Effective pair-potential, genome-wide classification, and compressibility.* **Panel A**: Shown is the result for *C. albicans*. Each chromosome is partitioned into several sections, each containing 50, 000 base pairs with two additional 12, 500 bp intersections on both sides. The curves are the effective potentials, which quantify the global interaction pattern between nucleosomes. Their coloring is adjusted to be consistent with panel B. **Panel B** shows the classification of the sections based on the pair potentials and compressibilities for the whole genome. This classification is based on a k-means clustering into 3 clusters. They are intentionally classified to be comparable with the classification of heterochromatin, euchromatin, and differently organized. The dashed lines are the compressibility results. The two yellow and the two blue lines mark the position of known characterization. **Panel C**: This panel shows the reduced isothermal compressibility $\chi _T^\infty$ employing the block density method. The plot displays the process for chr. 2. The x-axis is the number of blocks $M_b$. The linked dots are the compressibilities of block $\chi _T(L, L_0)$. By extrapolating their linear regressions, we obtain the intercepts as the compressibility, marked by triangles. **Panel D:** For a better representation of the complex structure, we calculated the distribution of the compressibility $P(\chi _T^\infty$)

For the differences between the target RDF and the simulated, we used the mean squared residual (MSR)

7 $$\begin{aligned} MSR = \frac{1}{(n-p)}\sum (x-\hat{x})^2 \; , \end{aligned}$$

where *p* is the number of parameters in the regression (including the intercept). *x* is the target value, and $\hat{x}$ is an estimator.

For the modified Lennard-Jones potential the domain of $\sigma$ is $\left[ 140,170\right]$. It has the unit of one base pair. Inside the ISS, the selecting ratio is 0.25.

### Classification

The resulting potentials from the Monte Carlo with its parameters can be used for clustering approaches such as k-means. Panel C in Fig. 1 shows the obtained values for the exponents as well as on the z-axis the compressibility data. The parameters $\nu$ and $\delta$ that characterize the short range repulsion and the long-range attraction together with the information on the compressibility are used for a k-means clustering.

## Results

### Effective potentials and classification

The results on the effective potential for *C. albicans* are shown in Fig. 2a. The colors indicate the class according to a k-means clustering based on three clusters taking into account the exponents and the compressibility (see Figs. 1 and 2 Panels C and D.) From Fig. 2a, it can be seen that they all share a minimum lying between 160 bp and 180 bp. However, the well depths are falling into different classes. A shallow minimum with a steep repulsive part indicates an area where nucleosomes are loosely bound, corresponding to an irregular array, i.e., with liquid-like structure. A deep minimum with a less steep repulsion leads to a regular array in contrast, i.e., a much more ordered structure. Thus, the section partitions into those that are liquid- and those that are more solid-like in agreement with the classical classification eu- and hetero-chromatin picture, disregarding the nuances of a finer partitioning. However, the classification did no trivially sort the potentials according to the potential minima. Rather, an interplay between attraction, repulsion, and compressibility can be seen. The sorting into classes is more toward how the potential behaves at short distances and a larger distances, whereas in the well part of the potential a substantial criss-crossing can be seen the far ends are much more sorted.

The classification is based on all of the sections of the entire genome. This effectively constraints the pattern to be of a universal genome-wide character. Local variations are subsumed into broader classes filtering out the universal patterns underlying the local variations within a chromosome as well as among the chromosomes.

The resulting coloring of three clusters is shown in Fig. 2b. The coloring of Fig. 2a is adjusted to be consistent with that in panel B. The classification results suggest that there is more than hetero- and euchromatin. At least a further class can be distinguished genome-wide. In the supplementary information, Figure S3 shows a principal component analysis for various given k-means clusterings. Since we cannot employ directly a method such as the elbow method to look for the best classification, the visual inspection partitioning of the clusters in principal component space is used. A classification into three clusters shows the best result. Two clusters show a trivial partition while for a larger number of clusters a significant overlap is seen. Indeed, already in the first experiments it was noticed that within hetero- and euchromatin variations exist [38].

The result of the classification into three classes mapped to their original genomic location is shown in panel B of Fig. 2. Also shown in the figure are the results for the compressibility. The compressibilities themselves are shown in panel C and D. In Fig. 2c, we show the results from the block density method for all sections in chr. 2. Each line presents one section. The linked dots are the reduced isothermal compressibility of block $\chi _T(L, L_0)$ with respect to the number of blocks $M_b$. The straight lines are the corresponding linear regression results for the extrapolation to the thermodynamic limit. The triangles mark the intercepts, i.e., the reduced isothermal compressibilities $\chi _T^\infty$. All lines are colored according to their $\chi _T^\infty$ value. Note that no corrections for the scaling are necessary as the extrapolation proportional to 1/*L* is consistent with the data.

The distribution of the extrapolated compressibility values for the whole genome (for *C. albicans*) is shown in Fig. 2d. The distribution is clearly non-gaussian. The obtained extrapolated values are used for the classification and shown in panel B. A high value of compressibility is associated with a few location along the chromosomes. Marked by the thick black line is the location of the centromeres. Four further markers from gene expression results confirmed by three experimental groups [23, 39, 40] are also included. They have measured the expression for those genes in different conditions, especially in different iron concentrations, and they concluded that in our circumstance, the two blue marked regions were suppressed while the yellow marked regions were not suppressed. Both results are compatible with the classification. The sections that are classified as heterochromatin are indeed consistent with the deeper wells of the potentials while the euchromatic region is in general associated with more shallow wells of the potentials (Fig. 2).

## Conclusion

Based on the nucleosomal positioning data, the extraction of effective potentials is possible for an entire genome. If this information is supplemented with thermodynamic information in terms of compressibility, i.e., density fluctuations, a genome-wide consistent classification in sections is possible. The classification into the classes shows that at least three different classes must exist. Hence, beyond hetero- and euchromatin a third kind of ordering is necessary. The grouping of the exponents of the generalized Lennard-Jones potential may suggest that there may be more than three classes. However, the principal component analysis of the parameters into two dimensions shows that at least for this projection three is the best decomposition into classes.

Positioning data and simulations of the fluctuations of the positioning data should incorporate such effects as nucleation of hetero-chromatic regions. Thus, in a consistent manner the classification into more or less ordered regions is possible. Beyond this classification, having the information on the coarse-grained potentials, this approach allows for the modeling of chromosomes as hetero-polymers with inter-nucleosomal interactions. If this is further augmented with inter-chromosomal information derived from chromosomal conformation capture methods, a consistent framework for the simulation of chromosomes with the effective potentials is possible. This then allows to look for the mechanics, i.e., the mechanical code. Having the information on the potentials enables the modeling of the nucleosomes as effective disks such that the steric interactions together with the density fluctuations yield information on the stiffness of the particular section and thus on its bending rigidity.

One aspect of the ordering and stiffness of segments that is not yet covered by the approach are methylation effects. However, this can in principal be incorporated if a consistent set of experimental data would be available for a particular genome. This would add a further dimension for the classification.

## Supplementary Information

Below is the link to the electronic supplementary material.

Supplementary file 1 (pdf 474 KB)
