# Supplementary material for: Inter-nucleosomal potentials from nucleosomal positioning data
Source: Eur Phys J E Soft Matter. 2022 Apr 11;45(4):33. doi: 10.1140/epje/s10189-022-00185-3 (PMC9001623; doi:10.1140/epje/s10189-022-00185-3)
Supplement: Supplementary file 1 — (pdf 474 KB) [file 10189_2022_185_MOESM1_ESM.pdf]

Supplemental Information

The source code for the program is available at https://github.com/mdscolour/reverseMC.

The genome-wide effective potential data as well as the corresponding compressibility is available at the following DOI link: https://doi.org/10.11588/data/H3KPEU.

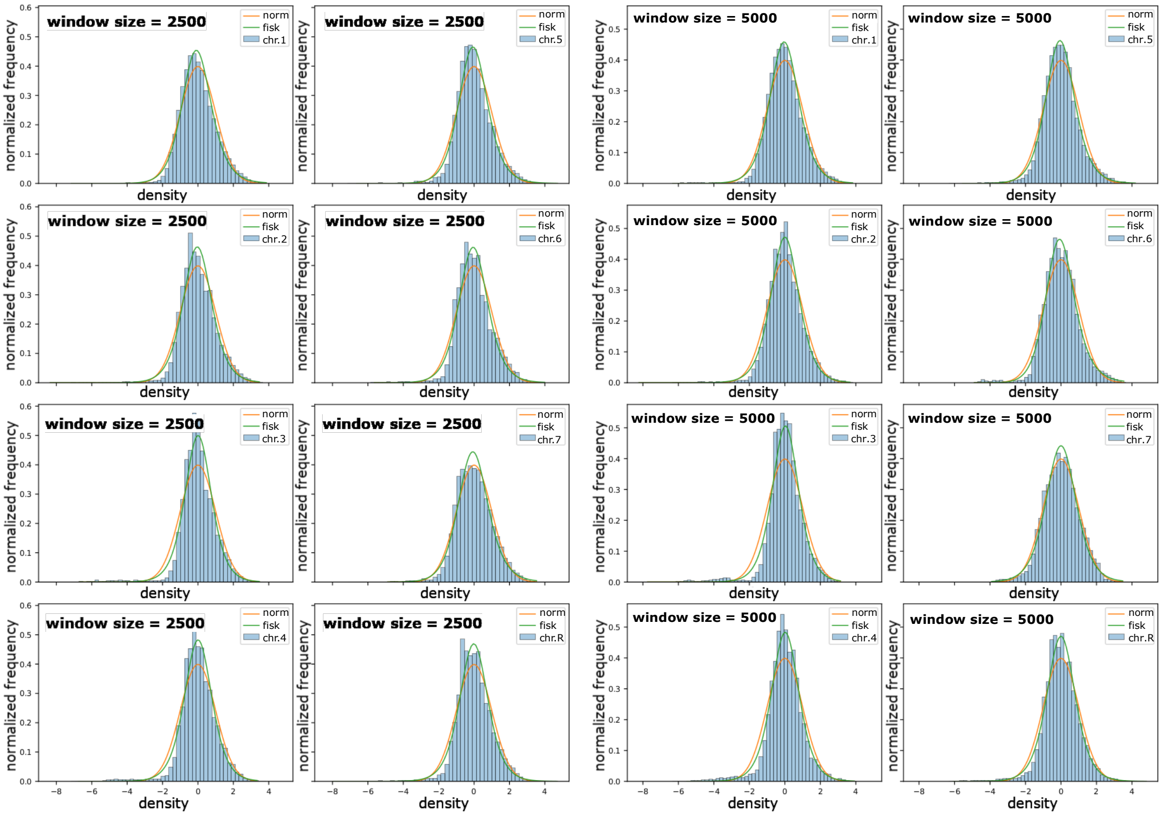

Figure S1: **Nucleosomal density after coarse-graining** The nucleosomal density distribution appears to be close to a Fisk distribution, i.e. is a log-logistic distribution. Shown are the results for a window size of 2500 and 5000. Several window sizes are examined and the 5000 bp length is the most suitable coarse-graining scale. Hence the typical section length is chosen to be 50000 bp.

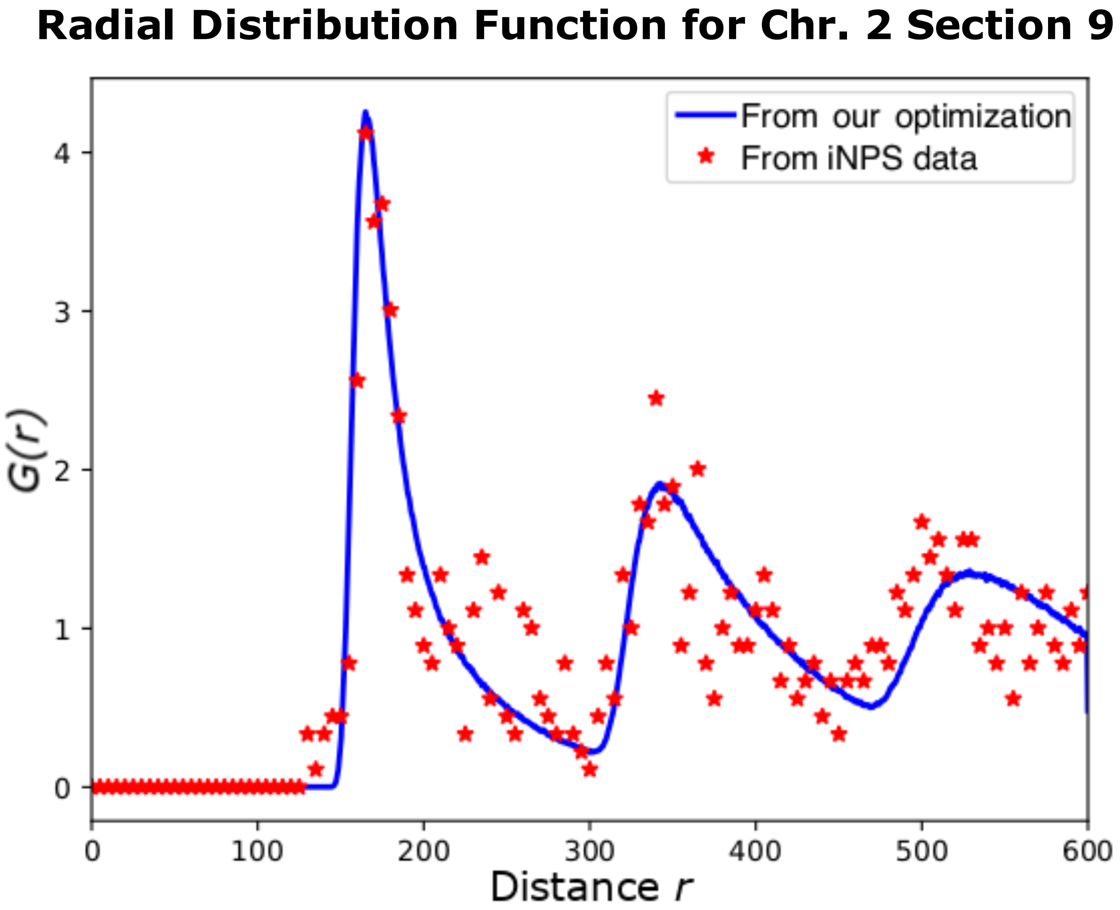

Figure S2: **iNPS data and resulting radial distribution function for chr. 2 section 9** Red stars show the radial distribution function (RDF) data calculated from experimental iNPS data. The blue curve is the estimated result for the effective potential at the same area by implementing An MC simulation. The RDF is computed from a total of 150000 MC steps.

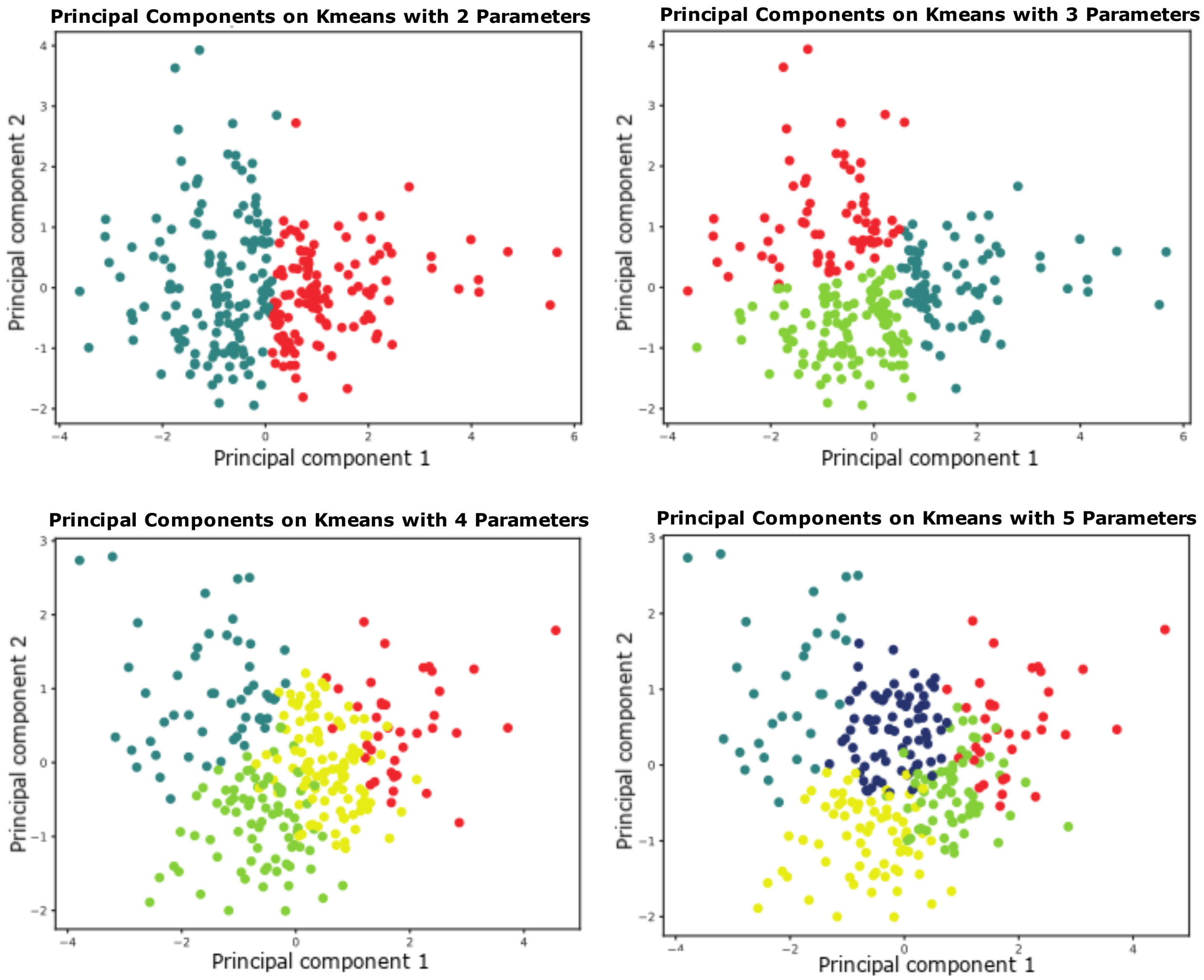

Figure S3: **PCA on K-means** Shown are the principal component analysis for a two-dimensional projection of the k-means clustering with various given cluster numbers. Rather than using the elbow or similar methods to find the optimum number of clusters, we have chosen to visually detect the best number of clusters. From the visual inspection we see that two clusters trivially separate into two clusters. The cluster separate non-trivially for three clusters whereas, above three clusters there is always a non-negligible overlap between the clusters. The parameters for the k-means clustering were ν, the potential minimum and the compressibility χ within the section.
